# Recognizing and Mitigating Canine Stress during Animal Assisted Interventions

**DOI:** 10.3390/vetsci8110254

**Published:** 2021-10-27

**Authors:** Lisa Townsend, Nancy R. Gee

**Affiliations:** 1 Center for Human-Animal Interaction, Department of Pediatrics, Division of Adolescent Medicine, School of Medicine, Virginia Commonwealth University, Richmond, VA 23298, USA; 2 Center for Human-Animal Interaction, Department of Psychiatry, School of Medicine, Virginia Commonwealth University, Richmond, VA 23298, USA; Nancy.Gee@VCUHealth.org

**Keywords:** animal-assisted interventions, animal-assisted therapy, animal-assisted activities, therapy dogs, canine stress, canine agency, intervention characteristics, stress mitigation

## Abstract

Animal-assisted Interventions (AAI) proliferated rapidly since clinicians and researchers first noted the positive effects animals have on people struggling with physical and mental health concerns. The intersection of AAI with the field of animal welfare evolved from considering animals’ basic needs, such as freedom from pain, to recognition that animals experience nuanced emotions. Current conceptualizations of the various roles of companion animals as an adjunct to treatments for humans emphasize not only the animals’ physical comfort and autonomy, but also their mental well-being and enjoyment of AAI activities. However, numerous challenges to effective monitoring of animals involved in AAI exist. This article focuses specifically on dogs, highlighting factors that may lead handlers and therapists to miss or ignore canine stress signals during human-animal interactions and offers strategies to recognize and ameliorate dogs’ distress more consistently. The primary goals of this discussion are to summarize the current thinking on canine well-being and to highlight practical applications of animal welfare principles in real-world AAI settings. The paper highlights contextual factors (e.g., physical setting, patient demand), human influences (e.g., desire to help), and intervention characteristics (e.g., presence or absence of a dog-specific advocate) that may promote or inhibit humans’ ability to advocate for therapy dogs during AAI activities. Deidentified examples of each of these factors are discussed, and recommendations are provided to mitigate factors that interfere with timely recognition and amelioration of canine distress.

## 1. Introduction

In this paper, we describe the multiple contexts in which animal-assisted interventions (AAI) take place, as well as the numerous challenges to effective monitoring and response to canine stress that handlers and providers face. We summarize current evidence regarding canine emotions, cognitions, and stress signaling, and highlight factors that inhibit timely detection of canine stress. We provide case examples that contextualize these factors in “real world” scenarios and provide practical strategies for alleviating canine stress and enhancing dogs’ enjoyment of animal-assisted interventions.

Humans have long known the benefits animals can have on physical and psychological recovery. During the Roman Empire, healers noted that the presence of animals uplifted people who were suffering; Florence Nightingale and Sigmund Freud observed similar dynamics between animals and their patients [1]. Animal-assisted interventions and research regarding their application and efficacy proliferated following Boris Levinson’s description of the beneficial effects of his dog, Jingles, on his therapeutic work with children [2].

## 2. Terminology and Settings

Knowledge regarding the characteristics of and settings in which animal-assisted activities take place is essential to ensuring the welfare and comfort of animals who participate. Additionally, the use of a single, agreed set of terms allows for clear communication and transparent sharing of information. The International Association of Human-Animal Interaction Organization (IAHAIO) defines *Animal-Assisted Activities* (AAA) as “informal interactions/visitations often conducted on a volunteer basis by the human-animal team for motivational, educational, and recreational purposes.” Activities are conducted by individuals who are not required to have education in health or human services and there is no specific intervention goal that guides the interaction [3]. In contrast, *Animal-Assisted Therapy* (AAT) “is a goal oriented, planned, and structured therapeutic intervention directed and/or delivered by health, education, and human service professionals.” Specified intervention goals guide the interactions, and client progress is documented [3]. *Animal-Assisted Education* (AAE) “is a goal oriented, planned and structured intervention directed and/or delivered by educational and related service professionals.” AAE activities further academic, social, or cognitive goals [3]. *Animal-Assisted Crisis Response* (AACR) teams undergo specialized training and screening for suitability to intervene during crises, which can be characterized by environmental unpredictability and emotional intensity [4]. National standards were developed to ensure that handlers and dogs meet minimum standards for training, health, and well-being [5]. Each of the settings described above (AAA, AAT, AAE, and AACR) are characterized by unique goals and attributes that directly impact animal well-being as well as pressures that influence handler recognition of stress signals from their dog.

### Evaluating Therapy Dogs

AAI organizations strengthened their emphasis on assessing whether an animal enjoys interacting with people and participating in animal-assisted activities. IAHAIO formally incorporated animal well-being into their guidelines, noting that AAAs “should only be performed with the assistance of animals that are in good health, both physically and emotionally and that enjoy this type of activity” [3]. At least two major U.S. therapy dog registration programs (Pet Partners and Alliance of Therapy Dogs) now include animal willingness to engage in interactions with humans as an essential element of their therapy dog evaluations. Pet Partners’ training program underscores the YAYABA (“You are Your Animal’s Best Advocate”) concept, which symbolizes the vital role that handlers play in providing for their animals’ well-being at all times during animal-assisted activities [6,7]. The concept of “goodness of fit” has been applied to the process of choosing dogs with specific temperaments, preferences, and energy levels that are aligned with the contexts in which animal-assisted activities occur [8]. To best evaluate a therapy dog’s willingness to engage in interactions with others, we need tools to evaluate their willingness to be involved.

## 3. Advancements in Animal Welfare: From Basic Needs to Nuanced Emotions

As the role of animals in therapeutic work with humans expanded, provisions for the welfare of animals engaged in AAI became increasingly necessary. Early focus on animal welfare considered only the basic needs of livestock (freedom from hunger/thirst, discomfort, pain/injury/disease, fear and distress, and the freedom to express normal behavior) [9]. Advances in understanding of animal cognition and behavior prompted recognition that dogs are capable of complex reasoning [10] and experience complex emotions [11], including empathy [12]. Acknowledgement that dogs experience an array of emotions must catalyze efforts to ensure that therapy dogs are not simply “used” as tools for service delivery, but instead are treated as sentient beings who experience the advantages and disadvantages of their therapeutic roles similar to their human counterparts.

## 4. Recognizing Canine Stress, Enjoyment, and Enrichment

A primary role of the therapy dog handler is to monitor and provide for the animal’s well-being. This involves simultaneously monitoring canine stress signals, maintaining awareness of the environment, creating circumstances the dog finds rewarding, and interacting with people who receive animal-assisted services. Dogs communicate stress in a variety of ways, and as described below, some of that communication is extremely subtle. Generally, dogs progress from milder indications of distress, such as paw lifting, to more obvious signs that can include growling, snapping, or biting [13]. Expressions of stress can be unique to every dog, necessitating that handlers know their animals well and take the time to familiarize them with the therapy environment. Scientific evaluations of canine stress must be multimodal, evaluating multiple behavioral and physiological parameters when possible [14].

Although scientific understanding of canine enjoyment is nascent, mounting evidence suggests that dogs experience and communicate pleasure. Signs that a dog finds an activity or environment enjoyable include a relaxed posture, open mouth, and movement toward the interaction or activity [15]. Biological studies demonstrate improvements in neuroendocrine parameters (increases in plasma β -endorphin, oxytocin, prolactin, phenyl acetic acid, and dopamine) and blood pressure in both humans and dogs following brief, positive interactions [16], suggesting that interactions between humans and dogs can be associated with emotional and physiological benefits for both species.

As understanding of canine cognition and emotions has grown, attention has increasingly turned toward enriching the lives of our canine companions. Neurobiological evidence indicates that enrichment is associated with decreased hippocampal neuron loss in aging dogs [17]. Enrichment was also associated with increased social responsiveness and decreased problem behaviors in shelter dogs [18]. Enrichment ideally should include a wide array of social and sensory experiences, including novel activities, interactions with people and other conspecific animals, as well as taste, olfactory, and auditory sensations [19].

## 5. Canine Stress Signals

Dogs exhibit a variety of behaviors that communicate stress and distress. Interpretation of these stress signals is paramount to adequately prepare and then advocate effectively for the well-being of dogs involved in AAI. Studies of dog owners indicate that humans frequently miss subtle signs of canine stress even in their own dogs [20]. Interpretation of canine stress signals can be clouded by a number of factors, including breed-specific morphological differences in facial and body features, handler familiarity and relationship with a particular dog, and individual differences in how dogs are reinforced or aversively conditioned for expressions of distress. In addition, context matters. The meaning of a yawn may differ dramatically when it occurs near bedtime in a dog’s home than when it occurs during an AAI session [21]. Timely and responsive advocacy requires that humans maintain familiarity with the communication patterns of the breeds with which they are working, their dogs’ normative behavior, and how their dogs communicate distress. Early stress signals can be quite subtle, necessitating that handlers remain attuned to their dogs throughout all animal-assisted interactions. We will briefly highlight some, but not all, of these nuanced stress signals in dogs below:

### 5.1. Avoidance

Dogs who feel hesitant or uncertain about a situation demonstrate subtle indicators of avoidance. These include looking away, lifting a paw, exerting pressure against the handler, or actual hiding [15]. Subtle displacement behaviors like sniffing or yawning are frequently ignored or misinterpreted by handlers. During a human-animal interaction in a busy environment, avoidance and displacement signals may go unnoticed, disregarded, or actively discouraged by the handler, to compel the dog to engage with the human.

### 5.2. Fear

Dogs who progress beyond avoidance into fear exhibit more dramatic, recognizable stress signals. These may include elements of avoidance such as looking away and leaning away from the interaction, as well as additional physical signs such as bracing their hind legs, flattening their ears, lowering their heads, and tucking their tails [15]. If dogs are unable to obtain relief from the feared stimulus and feel trapped, they may progress toward displays of aggression [15].

### 5.3. Gaze

Dogs subtly communicate with humans via directed gazing and will seek information from their owners and even strangers regarding the safety of ambiguous stimuli [22]. Behavioral ratings suggest that dogs with insecure attachment to their handlers may gaze more at them during mock AAI sessions than dogs with secure attachment, although these findings were significant only at the trend level [23]. Other studies demonstrate that therapy dogs’ gaze is preferentially directed toward their handlers during therapy sessions [24]. Further research is needed to appreciate the communicative role of canine gaze more fully, and it is possible that gaze intent and duration may vary across dog breeds. For example, Border Collies were bred to herd flocks of sheep and it is believed that they stare at individual sheep as a form of intimidation to encourage the sheep to move in a specific direction [25]. In a study examining gaze duration in three breeds of dogs, Retrievers were found to gaze significantly longer at human faces than German Shepherds or Poodles [26].

### 5.4. Freezing

Dogs may freeze when they attempt unsuccessfully to avoid a threatening situation. Freezing is characterized by postures that reduce body size and protect vulnerable areas of the body. One goal of freezing is to minimize visibility; another is to deliver a calming signal to other beings, animal or human. These behaviors can be viewed as last efforts to avoid conflict and frequently precede overt aggression [27,28]. Freezing is often accompanied by difficulty responding to environmental cues, including handler commands [27].

### 5.5. Aggression

Aggressive behavior itself might seem self-evident; however, initial displays of defensive aggression in dogs can go unnoticed in a busy context and may not be recognized easily by humans [29]. Initial signs of aggressive arousal include raised hackles, closed mouth, alerting (attending to the stimulus), and targeting (staring at a chosen stimulus) [15]. Left unaddressed, the circumstance could progress to overt aggression, which includes growling, barking, lunging, air snapping, and even biting [30].

### 5.6. Animal Assent/Dissent

Although dogs cannot verbally express their consent to interact with humans, they do express “assent and dissent” [30] through body language and behavior. Unfortunately, many humans assume that dogs welcome physical touch and interaction and misread signs of dissent. The “canine consent test” [31], suggests that humans approach dogs using a nonthreatening, sideways stance while gently offering an open palm near the animal’s chest. A dog who remains motionless or moves away is communicating dissent. One who moves toward the proffered hand and initiates physical contact is communicating willingness to be touched. This consent test provides a useful method for determining whether a dog is willing to interact with people at a given time. Handlers can advocate for their dogs by gently instructing others about how best to approach them. Allowing dogs to provide consent and honoring their desire to dissent and move away from an interaction strengthens the handler-dog bond and models positive relationships for clients participating in the interaction [32].

## 6. Contextual Factors That Contribute to Stress in Dogs

### 6.1. Physical Setting

#### 6.1.1. Crowds

Dogs performing AAI are often asked to navigate environments in which many people are present. Even when they are conducting one-on-one interactions (e.g., in a private therapy session), they may be asked to walk through or wait in a crowded waiting room beforehand. Dogs in busy hospital environments may spend time in bustling corridors or in elevators with multiple people squeezed into a small space. Crowded areas typically give rise to elevated noise levels and may be characterized by balloons, music, small children moving quickly, emergency responders, and many other stimuli. Crowded locations are often accompanied by more traffic or construction on the drive to the location and/or near to the destination bringing additional sounds (truck or bus engines, horns, etc.), smells (exhaust fumes), and delays (creating pressure to hurry).

#### 6.1.2. Classroom

Educational settings often involve multiple people, including children of various developmental ages and abilities. Children’s behavior can be loud and spontaneous, especially if they are young. Dogs participating in AAE are often the central feature of a learning activity and can be surrounded by children who all want to interact with the dog at the same time. Many children were not taught the appropriate way to interact with a dog, making the dog vulnerable to rough handling such as ear- or tail-pulling, hugging, or other behaviors likely to stress the dog. Very young children may not understand that dogs should not eat puzzle pieces, or that legos should not be inserted into the dog’s ear, or that touching the dog in a sensitive area could be painful or startling to the dog.

#### 6.1.3. Hospital

Dogs who serve in hospital environments may be exposed to a variety of sensory experiences such as smells associated with bodily fluids or cleaning agents, sights and sounds of medical equipment, rapidly moving health care workers, throngs of people, and humans displaying intense emotions. It is possible that medication may accidentally be dropped on the floor, or a patient’s meal may be served while the dog is in the room. Circumstances may change rapidly, escalating from a quiet, calm interaction to intense, urgent activity. Many hospitals are located in dense, urban environments, necessitating that the dog walk along crowded city streets near heavy traffic, the hectic movements of people, and construction.

#### 6.1.4. One-to-One Visit

Visits with individuals may offer a quieter, calmer environment for human–animal interaction; however, these visits are characterized by their own unique stressors. A therapy dog may be confined to a small room or office space with a closed door which creates a feeling of confinement. Dogs may be exposed to atypical physical behavior and the expression of intense emotions depending on the client’s presenting problem. In some cases, clients may attempt to hug and hold the dog while they express their emotions, but this behavior may be alarming and uncomfortable to the dog.

#### 6.1.5. Noise and Urgency Level

Different therapeutic environments operate at different paces. A quiet, one-on-one therapy session might be slow-paced and calm, with little sense of urgency. In contrast, a busy hospital environment is characterized by sudden, fast-paced activities, such as staff responding quickly to a medical emergency.

### 6.2. Canine Characteristics

#### 6.2.1. Fatigue

Dogs’ tolerance for stress may change depending on the activities in which they engage and how physically and emotionally taxed they feel. Many organizations do not impose time limits on visit length, while others limit duration to one or two hours [30]. Fatigue affects many aspects of canine functioning, including cognition and frustration tolerance. Extending visits beyond a dog’s fatigue point can impair their ability to respond to cues and tolerate frustration.

#### 6.2.2. Aging

A dog’s age may differentially impact their response to therapy situations. An older dog may be more accustomed to the sights and sounds of a therapy environment; however, he or she might become more easily fatigued. In comparison, a younger dog may feel more stressed by the novelty of the therapy environment but will likely be more resilient to fatigue.

#### 6.2.3. Physical Health

A national survey of therapy dog organizations demonstrated that the majority require regular veterinary checks for registered dogs as well as recommended health checks if an animal exhibits signs of illness [30]. Advancements in illness identification began to incorporate observations of subtle animal communication. For example, animals experiencing the onset of illness are known to change posture and to selectively inhibit some feeding and social behaviors before clinical symptoms become apparent. These behavioral indicators are predictive of illness onset in many species [33]. Maintaining routine veterinary wellness checks combined with awareness of these subtle illness indicators in dogs and in a handler’s own dog offer the opportunity to proactively advocate for canine well-being in the context of AAI.

#### 6.2.4. Workload

The amount of time spent working in an AAI setting exerts a strong influence on a dog’s stress tolerance. If a dog has worked significantly more than usual, he or she may be more likely to exhibit signs of stress during an AAI interaction. Workload is especially relevant for dogs involved in AACR, which can involve travel to disaster sites and a prolonged period of intense interaction spanning several days [34]. Handlers need to closely monitor their dogs for signs of stress and establish a workload that is well within their own dog’s tolerance. It is also critical to continually monitor the dog for any signs that the current workload may have become too challenging so that the workload can be adjusted to better meet the dog’s needs.

#### 6.2.5. Trigger-Stacking

Trigger-stacking occurs when a dog undergoes multiple stressors without adequate return to baseline. This phenomenon can lead to the appearance of “unpredictable” behavior, such as growling or refusing to engage in an activity; however, many canine stress-related behaviors can be anticipated with thorough understanding of their antecedents. Trigger-stacking can occur over short or extended periods of time, depending upon the intensity and duration of the stressor [35,36]. For example, a dog who travels to an unfamiliar location for a family vacation may experience many stressors that are simply part of the fun for his or her humans. Depending upon whether car rides, changes in daily routine, water sources and unfamiliar housing, and people are stressful to a specific dog, he or she may return home unprepared to transition into an AAI visit right away. Conversely, a dog who has just spent an hour listening to the beep of a smoke alarm with a low battery may struggle to participate in an AAI session that would normally be easy for him or her. Physiological stress responses and return to baseline differ for each dog, making it imperative for handlers to maintain awareness of environmental conditions their dogs face not only the day of an AAI session, but also in the days leading up to that visit.

## 7. Intervention Characteristics That Contribute to Missed Stress Signals

The specific nature of an AAI can contribute to, or alleviate, stress and the ability of a handler to detect it.

### 7.1. Presence-Absence of Canine Advocate

#### 7.1.1. Diamond Model

The “Diamond Model” of AAI specifically provides for a handlers’ involvement in a therapy interaction [37]. Model components include the dog, a handler, the clinician, and patient(s). The handler assumes primary responsibility for the dog’s welfare during the session and collaborates with the therapist to ensure that therapeutic activities do not stress or harm the animal. The clinician assumes primary responsibility for the client. By design, this model includes a person whose primary responsibility is to ensure the welfare of the therapy animal and advocate for changes in the therapeutic interaction that address the animal’s well-being.

#### 7.1.2. Triangle Model

In the Triangle model, which is often employed in outpatient psychotherapy, the clinician is responsible for the simultaneous well-being of both dog and client [37]. This arrangement requires increased vigilance on the part of the handler/therapist given that he or she must continuously monitor their dog and the client for signs of distress. It is likely that canine stress signaling may be missed more frequently in the triangle than in the diamond model given the role of divided attention and potentially conflicting priorities for the therapist.

### 7.2. Intervention Complexity

Variability in the complexity of animal-assisted interventions is well documented in the literature [38]. Interventions vary widely in the degree of animal involvement, from simply asking the animal to be present to asking the animal to navigate obstacles or follow instructions. Individual differences in canine preferences become relevant given that some dogs may prefer simple, relaxed activities and others might prefer more active engagement and mental activity. Physical and cognitive overstimulation or boredom may all function as stressors.

### 7.3. Schedule and Frequency of Dog Involvement

The demands placed on therapy dogs vary considerably depending upon the client population, the setting, and interaction goals. Facility dogs accompany their handlers to work daily and typically remain onsite for eight hours. Other dogs may conduct hospital visits once or twice weekly, whereas some dogs only work occasionally. A study that examined canine cortisol production in response to human-animal interactions of varying durations and intensities found that salivary cortisol concentrations were higher on therapy days than on nontherapy days and increased following therapy sessions of shorter duration [39]. Handlers are recommended to adjust the intensity, duration, and frequency of canine participation to minimize animal distress. For example, high-intensity sessions should include frequent breaks, and canine participation in these interactions should be limited in frequency. In contrast, dogs may be involved in longer low-intensity sessions with less frequent breaks incorporated throughout [40]. Individual dogs vary in their tolerance for therapeutic interactions, requiring handlers to know their dogs and judge their participation accordingly.

## 8. Canine Factors

There are inconsistencies among dogs as to what will initiate a stressful response, and even the same dog may respond differently over time.

### 8.1. Health Changes

The majority of therapy animal organizations mandate regular veterinary health checks for their registered dogs [41]. This typically involves yearly health checks and documentation of vaccination status. However, health conditions may arise between yearly exams, rendering it vital for handlers to monitor changes in their dogs’ attitudes and behavior. Relatedly, determining when a therapy dog should retire is fraught with strong emotion for handlers. There is no single set of criteria for therapy animal retirement decisions; recommendations include multifactorial health and behavioral observations of the animal [42].

### 8.2. Canine Variability in Stress Signaling

The degree to which dogs become stressed by conditions they encounter in their roles as therapy animals is dependent upon multiple factors, including breed type and genetics [43] and those described earlier (e.g., workload, trigger stacking), but it is important to keep in mind that all dogs do not follow the same pattern of stress signaling, which can complicate the identification of stress signals in individual dogs. Canine experience and communication of stress and frustration may be further modified by training [44]), with reward-based training resulting in improved canine adjustment and relationships with humans [45].

#### Training out of Stress Indicators

Dogs may inadvertently or intentionally become conditioned to accelerate stress signaling by skipping lower levels of communicative behavior and moving directly to more overt indicators [46]. Dogs who find that mild stress signals, such as sniffing, yawning, or paw lifting, are routinely ignored may resort to task refusal or growling in the face of a significant stressor. These behavioral choices may be inadvertently reinforced by handlers who only detect and respond to overt signs while consistently missing their dog’s early signals of discomfort. Some handlers may punish their dogs for demonstrating certain stress signals, such as pinning their ears back or raising their lips to show their teeth. This handler reaction may also lead a dog to accelerate their stress signaling, especially if the handler becomes motivated to relieve the animal’s distress as a response to embarrassing behavior, such as growling. In these examples, dogs are reinforced for skipping lower-level stress indicators and moving ahead to employ signals that promise more immediate relief. The solution to this problem is to take lower-level canine stress signals seriously and provide relief rather than reinforcing the dog for use of more dramatic, overt stress signals.

## 9. Human Influences

Just as individual dogs will vary in their responses to stressful environments, handlers are differently equipped to respond to those same circumstances.

### 9.1. Level of Canine Behavior Knowledge and Willingness to Act on Knowledge

Pet owners vary in their knowledge regarding dog behavior and appropriate responses to canine stress signals [41]. While handlers can be expected to demonstrate greater knowledge of canine behavior given the training they receive through the therapy dog registration process [6,7], people vary in their background familiarity with dogs and their attitudes toward management of canine stress. A handler with many years’ experience detecting and responding to their dog’s communications is likely to respond more quickly and effectively than someone who is embarking upon AAI work with their first dog. Similarly, a change in setting brings new challenges to handlers and their dogs, necessitating that both learn a new environment together. Handlers new to a setting may experience more divided attention than handlers who are more familiar with that environment, causing them to miss their dogs’ stress signals. Evidence indicates that many owners would benefit from educational efforts to improve their ability to recognize and react to signs of stress in their dogs [29].

### 9.2. Urgency of Problem

Handlers may experience situational demand to meet the needs of people receiving AAI. Two contexts that may exert such pressure include palliative care and crisis response. Those settings are characterized by intense emotional need on the part of service recipients and urgency. These characteristics may influence handlers to extend their dogs’ working hours and reduce the frequency of breaks.

### 9.3. Other Pressures

Programmatic and internal pressures may reduce handlers’ detection of and/or willingness to respond to their dogs’ stress signals. A therapist whose entire practice depends upon one therapy dog may fear disappointing clients if the animal is ill or overtaxed. A visitation or educational program with a small team of therapy dogs may place more demand on each dog to meet service needs than programs with more handler-dog teams. Humans involved in AAI typically care a great deal about the welfare of other people, increasing their potential to ignore or rationalize their dogs’ need for downtime.

Recognizing stress indicators and the circumstances and influences that can trigger them will improve the ability of handlers to react quickly and in their dogs’ best interests.

## 10. Specific Recommendations

Because reading through the indicators and circumstances described above may not be sufficient to complete a handler’s awareness of dog stress and the complicated environments in which it can arise, we offer a recommendation for an assessment tool that can be helpful in evaluating AAA settings, followed by several case studies illustrating stressful situations and offering resolutions.

### 10.1. LEAD Assessment

Risk assessment is essential to setting up AAIs for success and preventing stress and potential injury to human and animal participants. The LEAD (Lincoln Education Assistance with Dogs) Assessment Tool [47] serves as the current best practice standard for risk assessment in animal-assisted activities, including animal-assisted interventions, education, and therapy. The tool provides a comprehensive framework for evaluating potential risks to human and animal participants tailored to specific environments. Undergirded by a philosophy of compassion for all human and animal participants, the tool emphasizes the importance of modeling care and concern for the welfare of animals and advocating on their behalf when signs of stress are observed. The LEAD tool provides a structured framework for identifying and mitigating environmental hazards as well as assignment of mitigation responsibility to predetermined designees. The LEAD assessment framework is easily adapted for individual dogs in specific settings and encourages AAI program leaders, staff, and handlers to anticipate and prevent potential hazards to human and animal participants.

### 10.2. Case Examples with Specific Recommendations and Resources

The following hypothetical examples highlight the complex contingencies that influence canine stress and handler decision-making during animal-assisted activities. The vignettes represent an amalgamation of the authors’ observations of canine-human interactions in a variety of therapeutic settings. They are meant to provide food for thought and to prompt creative thinking about how to adapt activities with animals to mitigate challenges to their welfare.

#### 10.2.1. AAA

Henry and Pickles volunteer at the Shady Pines nursing home. Pickles is a 5-year-old French bulldog who enjoys visiting older adults. She particularly enjoys sitting on the residents’ laps and pouncing on squeaky toys they throw to her. Pickles’ usual behavior on visit days is to run to the door and turn in circles excitedly when she sees Henry gather her therapy dog vest and leash. One morning, Pickles walked sadly away from the door and laid down under the kitchen table when Henry brought out her equipment. Henry tried to motivate Pickles for several minutes before finally cajoling her with treats. Henry was anxious to get to Shady Pines because the residents and staff were celebrating a resident’s centenarian milestone that day. During the visit, Pickles seemed reluctant to engage with anyone, licking her lips frequently when she was picked up and placed in people’s laps. She refused to chase her squeaky toys when they were thrown. Henry felt disappointed that Pickles was not engaged in the birthday celebration and worried that the guest of honor would be upset. Over the following two days at home, Pickles began moving stiffly, holding her paw up, and developed difficulty jumping onto furniture. When Henry paid a visit to the veterinarian, he discovered that Pickles had a pulled muscle that required crate rest. Henry recalled that Pickles played vigorously with his friend’s new puppy the day before; he suspected she pulled her muscle then. After taking a two-week break from Shady Pines to recover, Pickles was back to her happy self when they visited again.

This example highlights the pressures handlers face to accomplish specific goals during an animal-assisted intervention as well as the subtlety of canine stress signaling. Henry feared disappointing residents and staff at Shady Pines. In addition, his assumption, which was usually correct, was that Pickles loves visiting the nursing home. The onset of Pickles’ muscle soreness was subtle, leading Henry to misinterpret or disregard Pickles’ unusual behavior.

Mitigation strategies in this situation include allowing Pickles to stay home for the day and continuing to observe her unusual behavior. Henry could have gone to the birthday celebration at Shady Pines and explained that Pickles was temporarily under the weather. When Henry observed Pickles’ lack of enthusiasm at Shady Pines, he could have shortened the visit, explaining that Pickles didn’t appear to feel well and needed to go home. Table 1 presents ways to mitigate stressors associated with a variety of animal-assisted activities.

#### 10.2.2. AAE

Carrie and her dog, Bentley, a 4-year-old Australian Shepherd mix, volunteer in a special education classroom for first graders with developmental disabilities. They have been working with the kids for about six months and Bentley knows the children well. A new student, Sam, is introduced into the classroom; Carrie and Bentley meet Sam for the first time when they arrive to help with the classroom reading group. Sam was diagnosed with Autism spectrum disorder and was working with his occupational therapist to develop ways of communicating his needs without raising his voice, banging objects, or throwing things. In addition, Sam struggles with novel environments and changes to his routine. Sam often responds to change with yelling and demonstrating his discomfort with physical outbursts. Carrie and the teacher begin the reading group by having the kids take turns reading to Bentley. Each child sits next to Bentley to read aloud and show him the book’s illustrations. When the time comes for Sam’s turn, he begins shouting and waving his arms and throws his book across the room. Bentley licks his lips, puts his head down, and leans against Carrie’s side. The teacher’s aide quickly distracts Sam by taking him to a small coatroom adjacent to the classroom, where he sobs loudly and rocks. The teacher signals for the reading group to continue and the next student seats herself next to Bentley, who remains pressed against Carrie with one paw slightly off the floor.

A number of teacher and handler pressures present themselves in this educational setting. Teachers face a plethora of demands on their time, including responsibility for multiple students simultaneously and accountability for ensuring that students achieve learning goals. Bentley is demonstrating clear indicators of stress in response to Sam’s outburst and continues to communicate hesitance to remain engaged in the reading group. His compliant, nonaggressive nature led him to quietly ask for Carrie’s support; however, Carrie faces her own set of pressures to remain engaged in the reading activity. She knows that most of the students adore Bentley and look forward to seeing him each week. Some of them disliked reading before they met Bentley, who functions as a strong motivator for students to read. A few of the students will not read aloud to a human, whereas they forget their fear and read enthusiastically to Bentley. Carrie fears that disengaging from the reading group could upset the students and the teacher or make them seem unreliable. She also does not want to stigmatize Sam for his behavior.

Opportunities for mitigating canine stress while furthering students’ educational goals exist in this and similar situations. Communication between Carrie and the teacher ahead of time could forewarn Carrie that Sam may not respond well to Bentley initially. They could develop an alternate plan for introducing Sam to Bentley. Additionally, the teacher could communicate with Sam’s occupational therapist so that gradual exposure to a dog could be incorporated into his treatment plan. When Sam began to exhibit distress that affected Bentley, Carrie could have suggested that Bentley take an exercise break outside for a few minutes before returning to the reading circle. From a programmatic standpoint, close collaboration between the teacher and handler could enhance canine well-being and student safety as well as provide enhanced learning opportunities for the students. Learning activities that combine skills acquisition and addressing canine and student safety could include recognizing the meaning of human and canine nonverbal communication and practicing how to pet a dog safely [48]. These concepts could be incorporated as a fun element of a social skills curriculum. Table 2 provides suggestions for addressing many stressors associated with educational environments.

#### 10.2.3. AAT

Melanie is a cognitive-behavioral therapist in private practice who works with adults struggling with depressive and anxiety disorders. Her co-therapist is a Yorkie called Rufus. Rufus comes to work daily with Melanie and is extremely popular with the staff. Melanie and Rufus were working with a client, Angie, whose recovery from major depressive disorder was progressing steadily. One morning, Melanie receives an anguished message from Angie letting her know that her daughter was killed in a car accident the previous day. Upon returning her call, Melanie encourages Angie to attend her therapy appointment as usual so that she can talk things through and develop a crisis plan. Melanie is primarily concerned about Angie’s history of considering suicide when she experiences distress and wishes to assess her safety. During the appointment, Angie cries uncontrollably and scoops Rufus onto her lap for comfort. Angie folds over Rufus, holding him gently, yet not allowing him freedom to move away. Melanie notices that Rufus displays a sideways look, also known as a whale eye, several times while yawning frequently. Although Angie’s hold on Rufus is gentle, he is not free to move away from her. Melanie considers what she can do to help Rufus while not appearing to take him away from Angie. After 10 min, Rufus impulsively jumps down from the therapy couch and scampers to Melanie’s side, where he lies down and watches Angie intently.

The emotional distress inherent in AAT settings can feel overwhelming and difficult to metabolize for dogs and humans alike. In this example, Melanie employs the Triangle model of AAT, in which she is solely responsible for Rufus’ and Angie’s well-being in the session. Advocating for a therapy animal in the face of intense client distress feels difficult, in part because it could seem alienating to the patient. Yet, Rufus’ well-being cannot be subjugated to Angie’s needs. Despite the conflicting needs exemplified in this therapy example, there are several strategies that could be seamlessly employed to benefit Rufus and Angie. Planning ahead is a critical element of all effective therapy sessions. Melanie could incorporate hypothetical scenarios into her session planning, especially anticipating patient distress. She could consider how she will deftly protect Rufus while simultaneously addressing the patient’s needs. One way she could have responded is to acknowledge Angie’s distress and calmly encourage her to change her body posture–for example, she could lean back against the backrest of the couch with open hands, palms facing upwards. This is a soothing posture for patients and would release Rufus from her “hug”. Alternatively, Melanie could sit next to Angie with Rufus on her lap; the comfort that comes from a supportive person sitting quietly beside a client cannot be underestimated. Given that Melanie received advanced warning that Angie would be distressed during the session, she also could have asked staff members to keep Rufus with them for the appointment. Table 3 offers ideas for mitigating stressors often encountered during animal-assisted therapy sessions.

## 11. Discussion

### 11.1. One Health

The One Health philosophy emerged 200 years ago with the recognition that humans and animals are similarly susceptible to certain diseases [49]. Informed by advancements in understanding about the interrelationships between human, pet, livestock, wild animal, and environmental well-being, the One Health model evolved to reflect that challenges to one sector reverberate throughout this interconnected system. Public health efforts led to improvements in the diagnosis and treatment of animal health conditions [50]. Attention has more recently turned to prioritizing the well-being of animals involved in human–animal interactions [51]. Broadly defined, the primary domains of therapy animal well-being were conceptualized as *physical* (freedom from hunger, thirst, pain, and discomfort), *natural* (expressing normal behavior and freedom of choice) and *affective* (adequate sleep and freedom from cognitive overstimulation and stress) [40]. Explorations of animal affective states suggest similar biological, neuroendocrine, and communicative underpinnings as humans [52], making it paramount to ensure that animals experience positive affective states when participating in human–animal interactions.

### 11.2. Mutual Benefit and Canine Agency

In keeping with the One Health model and current behavioral and neurobiological understanding of canine cognition and emotions, fulfilling our responsibility to our canine co-workers requires a stance that their welfare stands on equal footing with that of our clients. Generally, recipients of AAAs and AAIs choose to participate; our dogs do not. Winkle, Johnson, and Mills [53] extend the One Health model to promote animal agency in human–animal interactions; this includes tailoring animals’ work to include activities for which they are best suited in terms of capability, enjoyment, and agency and allowing them autonomy and choice regarding human-animal interactions. Viewing one’s dog as a therapy partner rather than a therapeutic tool builds the human-dog relationship and may promote safety and enjoyment for all participants in the interaction. [54]. This philosophy stands to mitigate one of the most significant pressures handlers face during AAA—that of disappointing clients. Winkle, Johnson, and Mills [53] argue that contrary to disappointing clients, privileging animal agency and autonomy models a foundation of compassion and caring that can only strengthen the handler/client relationship.

## 12. Conclusions

Human–animal interactions became more and more important in our lives and are increasingly recognized as having positive impacts on our health and well-being. Advancements in science gave us greater understanding of the ways in which dogs interpret their environment and the broad range of emotions they experience in response to their surroundings and interactions with humans. With greater understanding comes heightened responsibility to our canine companions and colleagues. Dogs are not “tools” to be used solely for the benefit of people; they are colleagues and collaborators who respond to stressors in many of the same ways that we do. They simply don’t communicate their feelings using the same language. It is incumbent upon those of us who work alongside dogs to understand the signals they send and represent their interests as they collaborate with us in improving human lives. This paper highlights some of the ways that dogs may become distressed in animal-assisted interventions and offers practical suggestions to prevent and mitigate canine stress. Key elements of canine stress mitigation are planning ahead for circumstances you may encounter with your canine collaborator and maintaining awareness of their responses during animal-assisted interactions. Advocating for our dogs is not only the best way to take care of our canine colleagues; it communicates empathy and kindness to our human clients as well.

## Figures and Tables

**Table 1:** Canine Stress Mitigation Strategies for Animal-assisted Activity Contexts.

Environmental Characteristics	Canine Stressors	Handler Contingencies	Mitigation Strategies
Nursing Home Visitation Hospital Visitation Employee Wellness Event University Student Stress Relief	Crowds High noise level Unfamiliar sounds Multiple people touching dog No pathway for animal to disengage or leave Slick floors Hot weather Hot pavement Multiple dogs present	High activity level Heightened attentional demands Crowd control Lengthy/indeterminate interaction time Desire to make patients/visitors feel better Pressure to interact/entertain Constant need to watch the clock	Thorough handler training in recognizing early signs of canine stress Frequent breaks to allow “escape” People on hand for crowd control and observation of dog Scheduling multiple dogs for shorter time frames and rotating them into and out of interactions Temperature mitigation techniques such as ice cubes or water Setting up interaction for success—i.e., treats to motivate dog to interact Setting timer to check in with dog/give them a break Monitoring how dog does at home after interaction; adjust future visits accordingly

**Table 2:** Canine Stress Mitigation Strategies for Animal-assisted Education Contexts.

Environmental Characteristics	Canine Stressors	Handler Contingencies	Mitigation Strategies
School/Classroom Pre-school Elementary school Middle school High school Gymnasium University Library Community meeting space	Small children Unpredictable movements and sounds Legos, Play-Doh, small toys that dogs could eat; things with wheels Balls of different sizes (some bigger than dog) Food and snacks that children want to feed them Unregistered dogs may have different levels of training and tolerance for AAE Slippery flooring Demands for continual movement Other classroom pets (hamster, gerbil)	Pressure to meet educational goal Crowd control Navigating unfamiliar environment Ever-changing environment (school hallways, classroom decorations) Divided attentional demands if handler is also the teacher	Proactive education about how to interact with dog Additional advocates to serve as “buffer“ Pre-event visit to familiarize dog with setting Plan in advance to clear the floor of debris Train children how to interact with dogs Provide space for those who do not want to interact with dog Involve a classroom aide if teacher is serving as handler

**Table 3:** Canine Stress Mitigation Strategies for Animal-assisted Therapy Contexts.

Individual/Group Therapy			
Diamond Model	Heightened emotional intensity Sights and sounds in waiting room No pathway for animal to disengage or leave	Pressure to meet therapeutic goal Possible reduced control regarding structure of session Reduced flexibility to change course of session	Communicate with therapist ahead of time in re: animal welfare Prepare ahead of time for how to incorporate animal in session Arrange area in therapy room for animal to take space Set up brief "visit within a visit" in which animal attends smaller part of session
Triangle Model	Heightened emotional intensity Longer work hours if dog spends the day with therapist No pathway for animal to disengage or leave	Back-to-back sessions/productivity requirements Sole responsibility to account for client and animal welfare in session Divided attention due to focus on client Treatment plan involves participation of dog	Rehearse hypothetical therapy scenarios that affect dog welfare and generate appropriate responses Prepare client ahead of time that the dog may require breaks Inform client that the treatment plan may involve activities without the dog Incorporate client into animal welfare check-in ("What do you sense about Fluffy right now? What might she be thinking?")

## Data Availability

Not applicable.
